# The Meditative Patient

**Published:** 1920-01-17

**Authors:** 


### THE MEDITATIVE PATIENT.

ONE is tempted to think that hospital patients must either be inarticulate or familiar with institutional life, so rare is any record of their experience. The fancy that they are inarticulate is not always removed when their books have been read. There is the "bright" little volume of hospital sketches; there is the soliloquy from a sanatorium. To one or other of these classes a book about hospitals is almost certain to belong; nor has the literature of the war-hospital really added a new category. To all readers, however, who enjoy M. Maerterlinck, the Arthur Benson of Belgium, the meditative patient has a charm, and he is before us again in Mr. Compton Leith's new volume "Domus Doloris." It is amusing to hunt through these elusive pages the thin thread of fact on which the meditations hang, but this thread is concealed so artfully that, after all, we may have missed it. It would seem, however, that the author has been for some time a patient in St. Bartholomew's Hospital, and has confided to Mr. John Lane the publication of his meditations. Readers of Mr. Leith's previous books will know what to expect: the sophisticated style, the literary allusions, the soothing sequence of words. By these means what impression would he leave upon us? It is the impression of admiration for a cheerful, disciplined life, the religion of which is translated into immediate action. The writer invests with his own sense of romance the night nurse, the day sister, the brisk, alert matron, and believes that these live in a world which is more bracing to the character than that of those who engage in their own affairs outside. The impression is flattering, but he hints—for clarity is not his strong point—that it may be insufficient. The book for which we still wait is one by a nurse of long experience who has the eye to see and to record the life that she has lived. She should be able to reflect every ripple on the stream, some eye for character, and the rare power of seeing the quickly shifting centre which was the life of the place from day to day. There is an authenticity in truth which gives edge to its expression, and yet, since the writings of Florence Nightingale, where is it to be found? Till such an observer has appeared we must be grateful for more superficial impressions. For she who lives in hospital, not the patient of passage, nor the surgeon of six months' residence, is the person whose experience is nearest the centre of institutional life.

